# Assessing the Preference of Rabbit Does to Social Contact or Seclusion: Results of Different Investigations

**DOI:** 10.3390/ani10020286

**Published:** 2020-02-12

**Authors:** Alessandro Dal Bosco, Alice Cartoni Mancinelli, Steffen Hoy, Melania Martino, Simona Mattioli, Elisa Cotozzolo, Cesare Castellini

**Affiliations:** 1 Department of Agricultural, Food and Environmental Science, University of Perugia, Borgo 20 Giugno, 74, 06100 Perugia, Italy; alessandro.dalbosco@unipg.it (A.D.B.); mela_martino@hotmail.it (M.M.); simona.mattioli@hotmail.it (S.M.); elisa.cotozzolo@libero.it (E.C.); cesare.castellini@unipg.it (C.C.); 2 Department of Animal Breeding and Genetics, Justus Liebig University, D-35390 Giessen, Germany; Steffen.Hoy@agrar.uni-giessen.de

**Keywords:** rabbit does, motivational cage, social contact, preference test, group housing

## Abstract

**Simple Summary:**

Many studies were developed to improve welfare of domestic rabbits and on the possibility to increase contacts between co-specifics using animal group housing. However, a lot of behavior disorder in rabbit does was reported. This study presents two experiments carried out, respectively, in Italy (experiment 1) and in Germany (experiment 2), to evaluate the does’ motivation towards social contact. The Italian trial was based on a choice of test cages to investigate the preferences of nulliparous rabbit does for contacts with co-specifics (in a group of four animals) or seclusion. The German trial tested a different group-housing system constituted of four does and their kits to evaluate the same behavioral aspect. Experiment 1 showed that the time spent in seclusion or in group was almost the same (49.61% vs. 50.39%, respectively) whereas, experiment 2 showed that female does with kits preferred to stay alone (71.90%) rather than in a group, probably due to a hierarchical response stimulation. Besides, it showed a different individual preference; not all does like to share space with the others. This study suggests the need to find a cage adapted to the different physiological phase of does, for example, with the possibility to modify it during the breed cycle.

**Abstract:**

The aim of this study was to verify the motivation of rabbit does to social contact or seclusion. The results of two different research activities assessed in Italy (experiment 1) and Germany (experiment 2) through the use of motivational cages are reported. In experiment 1, only the average time of occupation of the group or seclusion zone was recorded of four nulliparous does, while, in experiment 2, the group-housing system provided space for does with kits and consisted of four single areas (nest boxes with individual electronic nest box recognition systems). Experiment 1 showed that does spent a similar amount of time in seclusion or in group (49.61% vs 50.39%, respectively). On the contrary, in experiment 2, does with kits appeared to prefer spending time alone (71.90%) rather than in groups. The presence of kits probably stimulates a hierarchical and aggressive response of the dominant does, with the low-ranking does staying secluded to avoid violent interactions. In fact, in each reproductive cycle, one doe did stay in the group area whereas the other three does used this area in different percentages of time. Further researches are needed to find a good combination of the cage with the does’ physiological phases.

## 1. Introduction

Rabbits are social animals and should be allowed to express all their behavioral patterns when farmed. A previous study showed that domestic rabbits exhibited the same social and territorial behavior as the wild ones [1]. Accordingly, as in other animal species, avoiding natural behavior can induce boredom, stress, and stereotypies in rabbits [2]. Furthermore, consumers are strongly concerned about animal welfare and therefore asking for more friendly rearing techniques.

Many researches have been conducted to improve the well-being of domestic rabbits. In a recent review [3], the authors came to the definitive conclusion, when looking at group housing of rabbit does, that aggression and injuries dramatically decrease welfare and performance of does and kits. These reasons make it—at the moment—inadvisable to use a continuous group housing of does under commercial conditions. The problems in continuous group housing are the following: high number of nest visits and behavioral disorders, high kit mortality, problems in health control (e.g., coccidiosis), aggressive behavior when replacement of does occurs, higher costs of production [4]. Moreover, high kit mortality and pseudopregnancy [5,6,7] can be detected in these group-housing does. The body of literature stated that continuous group housing is possible only in the rearing period and up to the first litter, and concluded that continuous group housing of does with kits means unnecessary stress and lesions to animals [8]. To avoid these problems, part-time group attempts were developed in which does are individually housed from a few days before parturition until some days after or when artificial insemination is applied [9,10,11]. Also, in other species (pig) the sow and piglets prefer to stay away from the rest of the herd for at least the first week after birth [12]. Although the grouping of does in some phases of the reproductive process has shown some potential, their adoption in farm conditions cannot yet be recommended due to the persistence of aggression and injured does [10,11].

Individual housing, although precluding social contacts between animals, prevents fighting for defending the nests [8] as well as double littering, which occurs when two (or even more) does litter in the same nest, resulting in high kit mortality due to crushing of kits and/or improper suckling [6].

Before any changes can be proposed, robust research is necessary to determine the optimal strategies and management techniques, as well as the cage design and the equipment for the safe establishment and maintenance of hierarchies, in order to limit harmful and painful behavior among does reared collectively. Social contact in group-housed does can be considered hypothetically useful under a welfare point of view [13], but other aspects (need to rest, feel safe) have to be considered and may be critical in this system.

Based on these considerations, a better knowledge of the ethology of rabbit does could certainly lead to finding structural or managerial solutions capable of combining the needs of animal social contact with the “real” welfare status of the animals, as well as the economic expectations of farmers. This is the knot to untie; surely before devising technical solutions it is necessary to thoroughly understand the preferred habits of rabbit does. To determine whether a change to the housing improves animal welfare, a number of approaches have been proposed, including the observation of changes in physiology or behavior and the measurement of the animal motivation to use different resources.

Accordingly, the aim of this study is to verify the motivation of rabbit does (nulliparous or females with kits) to social contact or seclusion; in particular, two different experimental activities (Italian and German) on this topic are reported.

## 2. Materials and Methods

The experimental design of both experiments was performed following the EU Directive 2010/63/EU [14] for the protection of animals used for scientific purpose.

### 2.1. Experiment 1

#### 2.1.1. Animals, Housing, and Husbandry

The purpose of this experiment was to study the motivation of nulliparous does for social contact or single housing. The experiment lasted 32 days.

**Figure 1:**
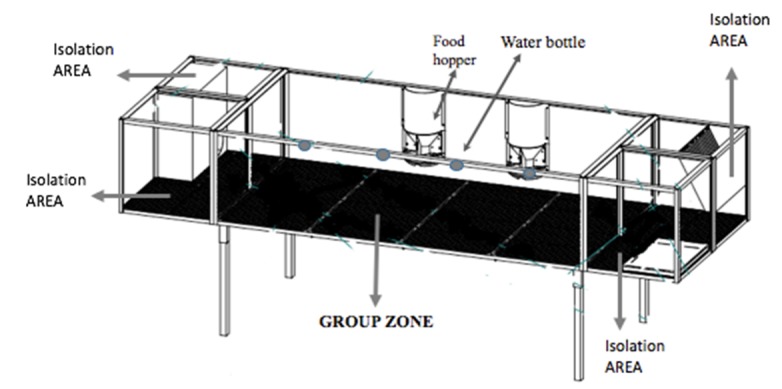
Scheme of motivational cage of experiment 1.

Eight nulliparous New Zealand White rabbit does (four per motivational cage) obtained from a commercial breeder at 13 weeks of age (live weight 2.8 kg) were used. The experiment was managed in the experimental farm of the Department of Agricultural, Food and Environmental Science of Perugia University, where the temperature ranged from +15 to +20 °C, relative humidity from 65% to 70%, and the photoperiod was 16 h light. The two motivational cages were designed by us and produced by Metac–Ellebi s.r.l. (Metac–Ellebi, Fabriano, Italy). Their width, length, and height dimensions were 76 × 250 × 60 cm and were equipped with four individual areas (38 × 25 ×35 cm) at two ends of the cage (see Figure 1). The group of young does was established one week before the starting of the trial.

The cages were constituted by:

- A group zone (located in the center of the cage) where the rabbits had the possibility to be in physical contact with the others;
- Four isolation areas connected with sliding doors where the rabbits had the opportunity to stay secluded.

The central zone (0.71 × 155 m and 0.61 m high) was equipped with feeders and drinkers and was surrounded by four isolation areas of the same dimensions. Each area could be reached from the central area through a one-way transparent Perspex push-door measuring 0.18 × 0.19 m.

As mentioned above, going through the push doors, the rabbit does could choose between spending time in group (social contact) or by themselves (seclusion in individual area). The rabbits had free access back to the group zone using the same door.

#### 2.1.2. Observation Method

Behavioral observations were carried out by a video-camera recording system, Noldus XT (innovative solutions for behavioral research). The video recording system was composed of a 4 to 9 mm varifocal lens, positioned on the pillars of the structure or on the beams of the roof from 3 to 5 m above ground level, so as to dominate the whole area used to house the animals. The cameras were connected, by means of a network of video cables and for power supply, to a video capture card inserted in a personal computer capable of transforming the digital analog signal for subsequent storage on a hard disk.

The analysis of the data consisted of screening video footage with software (Observer XT, Wageningen, the Netherlands) in which the operator set a specific coding scheme to rank the behaviors (e.g., the percentage of the time spent in the group or in isolation zones). Instantaneous sampling was the recording method used. We carried out video recordings of 120 min (60 min in the morning between 10:00 am and 11:00 am and 60 min in the afternoon between 14:00 pm and 15:00 pm). Based on our preliminary analysis, the intervals to estimate the percentage of time devoted to the various options was 5 s, repeated each 60 s.

### 2.2. Experiment 2

#### Animals, Housing, and Husbandry

**Figure 2:**
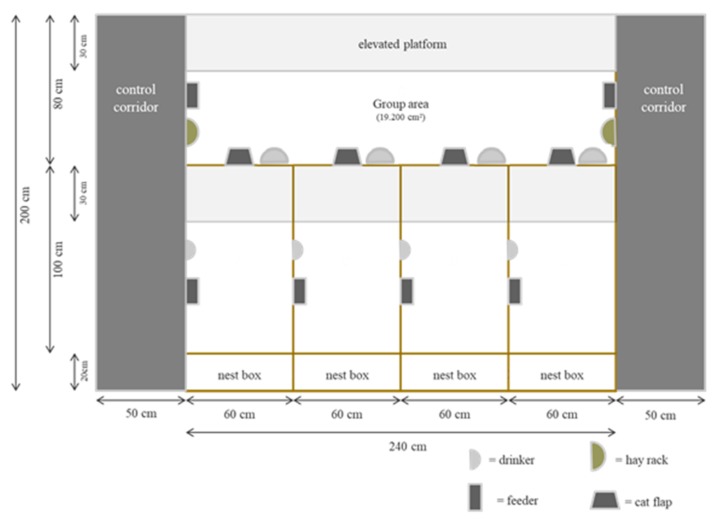
Scheme of group-housing system for does with kits of experiment 2 [16].

In the Department of Animal Breeding and Genetics of Justus Liebig University (Germany) an experimental group-housing system (Figure 2) was developed and some aspects of animal behavior investigated (i.e., eating behaviors, interaction between does like aggression and injuries, time spent in single or group area) [15].

**Figure 3:**
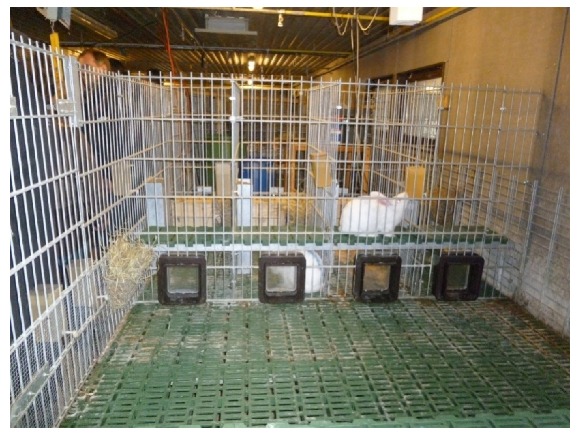
Group area and entrances to single areas through cat flaps of the experimental group-housing system for does with kits (experiment 2).

In experiment 2, the individual occupation of group or single areas was studied in does with kits. The group-housing system provided space for four New Zealand White does (150 d of age) with kits. After 35 days, the kits left the system and the does were reinseminated and started another cycle of production. The housing system consisted of four individual spaces (with nest boxes) with 6000 cm^2^ each according to the German Animal Protection Ordinance and a group area of 19,200 cm^2^. The issue of the free entrance of the does to their nest boxes was solved by using an individual electronic recognition system that allowed a doe to have access only to her own nest box. The special feature was the use of “cat flaps” (SureFlap) at the entry of the nest (Figure 3). The animals had a Radio Frequency IDentification microchip (FDX-B microchip), which made it possible for the does to get to their own assigned single area. The access from the individual to the group area, and vice versa, was kept free. Through these housing conditions, the does could decide freely where they wanted to stay: together with other does in the group area or alone in their individual area.

**Figure 4:**
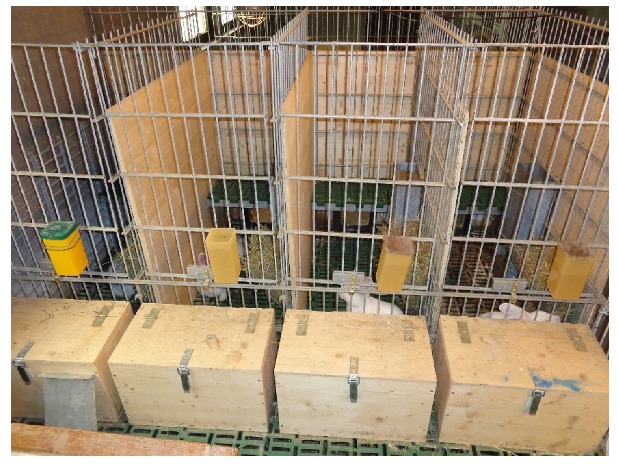
Single areas of the experimental group-housing system for does with kits (experiment 2).

The ground and the elevated platform were made from a commercial plastic slatted floor for piglets with 10 mm slat and 10 mm slot width. Both areas (single area, Figure 4, and group area) were provided with feeders, nipple drinkers, hay racks, and an elevated platform. Food and water were offered ad libitum.

A video camera (VK-1316S/12V, Panasonic Marketing Europe GmbH, Wiesbaden, Germany) and an infrared lamp (IRK-40/950, 12 W, Panasonic Marketing Europe GmbH) were installed above the group area. The behavior of the does was continuously recorded by time-lapse using a video recorder (CTR 4024, CBC Europe Ltd. London, UK), a monitor (WV-BM 990, Panasonic Marketing Europe GmbH) and 240 min video tapes. The videos were digitized using a specific software (Media Cruise Ver. 2.24, Canopus Co., Kobe, Japan). The files were analyzed by the program INTERACT (Ver. 9.0.7, Mangold, Arnstorf, Germany). The duration of the time spent in group or in the single areas was evaluated for each doe during 16 day cycles. A total of three cycles were analyzed.

### 2.3. Statistical Analysis

To compare the percentages of time spent in the single or group areas, the chi square independence test was used (experiment 1 and 2). Subsequently, the preferences for seclusion or group were analyzed with linear models comprising the effect of the hour of the day and motivation cage, repeated along time [17] (experiment 1) whereas, in experiment 2, the effect of cycle, and individual does and the repeated effect of time were analyzed. Means were compared using the *t* test and the significance was defined as *p* < 0.05.

Nonlinear regression (fractional polynomial regression) was also estimated to evaluate the time-dependent trend of the does (experiment 1). This procedure fits models with the best-fitting fractional polynomial.

## 3. Results and Discussion

The motivational tests are important to monitor the needs of the animals and positively contribute to their proper handling [18,19]. In the housing of rabbit does, there are two opposite trends to be considered; on one side is their need for social interaction and on the other our aim to ensure the well-being of the low-rank does, which are generally attacked by high-rank does [20]. However, it is not clear how the age, the reproductive activity, and the presence of kits affect the response of does to group housing.

Our results show that when nulliparous rabbit does (experiment 1) were free to cross the sliding doors, the time spent in seclusion or in group was almost the same (49.61% vs. 50.39%, respectively; Table 1). On the contrary, does with kits (experiment 2) showed a different behavior with a lower percentage of time spent in group (28.10%) rather than alone in the single cage (71.90%).

**Figure 5:**
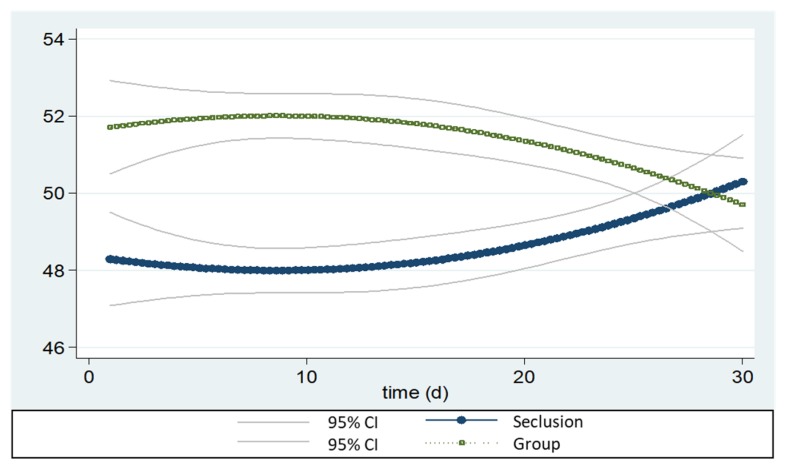
Time-dependent trend of time spent in groups or in seclusion for the rabbits (95% confidence intervals) in experiment 1.

Furthermore, experiment 1 showed that the choice of the does—between staying in group or seclusion—was not affected by the hour of the day (*p* > 0.05) nor by the group of does (e.g., motivational cage; *p* > 0.05); whereas there was a significant trend over time (*p* < 0.01). In fact, the time-dependent tendency of the seclusion significantly increases during the trial (Figure 5).

Moreover, experiment 2 showed that there was an individual preference in the use of different areas (Table 2). Each doe used the group/seclusion area in a different manner. Some does did not use the group area for one cycle, but did use it in other cycles, whereas the others used this area for different percentages of time.

A detailed analysis of the results and the comparison of the two experiments enabled us to show that the preference for seclusion or social contact in rabbit does was affected by several factors:

- Physiological state—the female does in different physiological states (nulliparous or during their reproductive functions) had a different response;
- Individual behavior—individual does had a different preference for seclusion or group;
- Presence/absence of feeders and drinkers—the presence of the feeders and drinkers in different areas of the cage could affect the use of resources;
- Video recording—the video recording system (continuous or not), the coding scheme, and the length of the recording probably had an effect on the result.

*a. Physiological State*

It is widely recognized that does become particularly aggressive during peripartum [21,22]. This trend is confirmed (experiment 2) by the comparison between the different amount of time spent in single cages or in group. The presence of kits probably stimulates the establishment of a stronger hierarchy and aggressive response of dominant does. Accordingly, low-ranking does prefer to stay secluded to avoid aggressive interactions. In some cases, the hierarchical establishment can lead to aggression, body lesions, prevention of conception, and infertility [23,24,25]. This hypothesis could not be directly evaluated with our experimental tools because the animals often avoided direct contact. During the same experiment, the does with kits did not show any body lesions, probably due to the presence of electronic identification, which prevented the access of aggressive does to nest boxes other than their own. With no electronic identification, previous trials [20] showed that high-ranking does, when close to kindling or having young kits, attack other does causing stress, fear, and body lesions.

Does with kits responded to the motivational test as follows: in one cycle, some were never in the group (probably the low-ranking ones) but they use it during other cycles, whereas others (probably the high-ranking ones) stayed in the group area for about half of their time.

On the other side, nulliparous does (experiment 1) did not show any aggressive behaviors and/or body lesions after the lag period for establishing a stable hierarchy. This confirms that when the does have no need to protect the litter, the behavior is more friendly. Accordingly, young rabbit does did not show any preference for seclusion or social contact.

In future research, it would be interesting to check whether young rabbit does, if weaned and reared in group, maintain a certain degree of friendly relations also during the reproductive cycle or whether the grouping during this phase nevertheless leads to the same aggressive effect [12].

*b. Individual Behavior*

In experiment 1, we estimated the time spent by the does in the different areas as an average and we had no results on the individual response of the animals. Conversely, in experiment 2, the individual behavior of the four different does was analyzed (Table 2). These results showed that the group area was not used by the four does in the same manner (*p* ≤ 0.05); in each cycle, one doe never used the group area whereas the others used this area for different percentages of time. The percentage of time spent in group of the other does varied between 5.6% (doe 2, in the third cycle) to 53.5% (doe 4, in the first cycle). In the last cycle especially, the does used the group area sporadically and preferred to stay in the single area. This increasing trend of seclusion is consistent with what was observed in nulliparous does.

As previously stated, the main reason of this behavior is probably a result of the ranking of the different does. Low-ranking does are stressed in the presence of higher-ranking does and prefer to be safe in the single cages.

*c. Presence/Absence of Feeders and Drinkers*

The presence of feeders and drinkers in the different areas could affect the occupation time of the motivational cage. It should be noted that about 10% of the time was spent by rabbits in eating and drinking activity [26], and thus the presence of feeder and drinkers placed in the group area only (experiment 1) could increase its occupation. Probably, the use of the feeder in combination with the group area (combined motivation), could affect the results. However, McGlone et al. [26] investigated the behavior of weanling pigs in pens equipped with hide areas. The study affirmed that the time spend by pigs on the feeder did not affect the access to the hide area. Future studies would be necessary with feeders present in all areas of the cages to avoid this possible bias and to enable the subject to feed easily.

*d. Type of Video Recording*

It is also possible that the video recording (continuous or not), the coding scheme, and the length of trial (16 vs. 32 days) affected the preference of the does. As an example, experiment 1 showed that there was a time-dependent trend on the use of resources (e.g., increase of seclusion). This trend is probably based on the instincts of the does, which experienced a higher comfort in single cages. The same was obtained in pigs, which, during the first 30 min after regrouping, used the hide areas to express the need of protection from attack [27].

## 4. General Conclusions

Based on these two experiments it is possible to draw some general conclusions. The motivational cages appear to be a suitable method to investigate the needs of rabbits for structures and contact with conspecifics. This methodology allows for testing the acceptance of various housing conditions and systems, and the assessment of new housing systems.

It has been established that nulliparous does behave differently from does with kits and that the latter ones are differently motivated to share a group area with other does. The cause of this diversity is probably the outcome of the hierarchy, where low-ranking does are stressed in the presence of higher-ranking ones. Accordingly, these results could be of practical relevance confirming that, with what is known at present, continuous group housing of does with kits should be avoided, given that no functioning housing system exists. During the reproductive period many does prefer staying alone and the low performance of these group-housed does has to be combined with other problems, like aggressive behavior, high kit mortality, health problems (e.g., coccidiosis), and higher costs of production. Without electronic identification and individual access to nest boxes, it seems impossible to guarantee the general function of continuous group housing of does with kits.

Probably, the group housing of does requires a flexible management of the group, restricting the group housing to some phase of the reproductive activity quite far from the peripartum period. Clearly, other experiments on group housing should be done to show if the motivation toward social contact increases or the preference for seclusion remains the same in different periods after kindling.

## Figures and Tables

**Table 1:** Percentage of time spent in groups or in seclusion for rabbit does in motivational cage.

**Experiment 1**	
Seclusion	49.61 ^a^
Group	50.39 ^a^
X^2^	2.10
**Experiment 2**	
Seclusion	71.90 ^b^
Group	28.10 ^a^
X^2^	10.30

^a,b^ for the same experiment and on the same column means *p* ≤ 0.05.

**Table 2:** Percent of time (%) spent in single or group area for the four does in cycles 1 to 3 (experiment 2).

	Cycle 1				Cycle 2				Cycle 3				SE
Doe	1	2	3	4	1	2	3	4	1	2	3	4	
Occupation time (%)													
Single area	100 ^b^	68.3 ^a^	47.8 ^a^	46.6 ^a^	50.0 ^a^	72.3 ^ab^	51.6 ^a^	100 ^b^	82.5 ^b^	94.4 ^b^	50.0 ^a^	100 ^b^	13.20
Group area	0 ^a^	31.7 ^b^	52.2 ^b^	53.4 ^b^	50.0 ^b^	27.7 ^ab^	48.4 ^b^	0 ^a^	17.5 ^a^	5.6 ^a^	50.0 ^b^	0 ^a^	8.34

^a,b^ on the same row for the same cycle means *p* ≤ 0.05.
